# Withdrawal: Ceramide stabilizes β-site amyloid precursor protein-cleaving enzyme 1 and promotes amyloid β-peptide biogenesis

**DOI:** 10.1016/j.jbc.2022.102052

**Published:** 2022-05-26

**Authors:** Luigi Puglielli, Blake C. Ellis, Aleister J. Saunders, Dora M. Kovacs

This article has been withdrawn by the authors. The Journal states that the immunoblots within panel H4 C105 in figure 2a were reused; the immunoblots in figure 5c and in figure 7a were reused. In addition, immunoblots in figure 7a and in figure 7b, within figure 7b, and within figure 7c panel corresponding to H4 751 were reused. Immunoblots portions within CHO 751 panel figure 6c were reused. Additional reuses are in figure 8a and 8b and within figure 8d. Due to the lack of original data, the authors wish to withdraw this article. The withdrawing authors stand by the overall conclusions of the study.

